# Advances and Perspectives in Prostate Cancer Biomarker Discovery in the Last 5 Years through Tissue and Urine Metabolomics

**DOI:** 10.3390/metabo11030181

**Published:** 2021-03-19

**Authors:** Ana Rita Lima, Joana Pinto, Filipa Amaro, Maria de Lourdes Bastos, Márcia Carvalho, Paula Guedes de Pinho

**Affiliations:** 1 UCIBIO/REQUIMTE, Laboratory of Toxicology, Department of Biological Sciences, Faculty of Pharmacy, University of Porto, Rua Jorge Viterbo Ferreira, 228, 4050-313 Porto, Portugal; jipinto@ff.up.pt (J.P.); famaro@ff.up.pt (F.A.); mlbastos@ff.up.pt (M.d.L.B.); 2 UFP Energy, Environment and Health Research Unit (FP-ENAS), University Fernando Pessoa, Praça Nove de Abril, 349, 4249-004 Porto, Portugal; 3 Faculty of Health Sciences, University Fernando Pessoa, Rua Carlos da Maia, 296, 4200-150 Porto, Portugal

**Keywords:** metabolomics, volatilomics, lipidomics, prostate cancer, urine, tissue, biomarkers, metabolic pathways

## Abstract

Prostate cancer (PCa) is the second most diagnosed cancer in men worldwide. For its screening, serum prostate specific antigen (PSA) test has been largely performed over the past decade, despite its lack of accuracy and inability to distinguish indolent from aggressive disease. Metabolomics has been widely applied in cancer biomarker discovery due to the well-known metabolic reprogramming characteristic of cancer cells. Most of the metabolomic studies have reported alterations in urine of PCa patients due its noninvasive collection, but the analysis of prostate tissue metabolome is an ideal approach to disclose specific modifications in PCa development. This review aims to summarize and discuss the most recent findings from tissue and urine metabolomic studies applied to PCa biomarker discovery. Eighteen metabolites were found consistently altered in PCa tissue among different studies, including alanine, arginine, uracil, glutamate, fumarate, and citrate. Urine metabolomic studies also showed consistency in the dysregulation of 15 metabolites and, interestingly, alterations in the levels of valine, taurine, leucine and citrate were found in common between urine and tissue studies. These findings unveil that the impact of PCa development in human metabolome may offer a promising strategy to find novel biomarkers for PCa diagnosis.

## 1. Introduction

Cancer diseases are one of the most important health problems worldwide, prostate cancer (PCa) being one of the most prevalent. Indeed, PCa is globally the second most frequently diagnosed male malignancy and the fifth leading cause of cancer, with more than 1,000,000 new cases and more than 350,000 deaths, each year [1]. PCa is a heterogeneous disease [2] with a broad spectrum of aggressiveness, going from indolent PCa, which is a non-life-threatening cancer, to metastatic PCa with a 5-year survival of 28% [3]. 

Currently, PCa screening is based in serum prostate specific antigen (PSA) test and digital rectal examination (DRE) [4], whereas prostate biopsy (PB) is mandatory for a final diagnosis [5]. High levels of PSA (>4 ng/mL) are considered a sign of PCa [4]. However, this biomarker shows important limitations [6], due to its reduced accuracy (accu) (62–75%) [7], sensitivity (sens) (20.5%), specificity (spec) (ranging from 51% to 91%) [4,8], and area under the curve (AUC) (varying from 0.53 to 0.83) [7]. These low performance values can be due to interference from other diseases, like benign prostate hyperplasia (BPH) or prostatitis, that may also lead to an increase in serum PSA levels [2,6]. Furthermore, PSA testing is unable to distinguish indolent from aggressive disease, leading to unnecessary PB [2]. As matter of fact, about 70% of the PB performed due to high levels of PSA do not detect PCa and could be avoided with a more accurate PCa screening test [6]. PB is an invasive procedure that is associated to several adverse effects, like hemoejaculate, hematuria, fever, pain, and hematochezia. Although more rare, other complications, like bleeding, acute urinary retention, local infection, sepsis, vasovagal syncope, and erectile dysfunction, can also occur as a consequence of PB [9]. Moreover, PB can fail to diagnose over 30% of clinically significant PCa (non-indolent). On the other hand, PB can also lead to overdiagnosis and overtreatment of indolent PCa, that will not bring advantages to the patients′ health and can negatively affect patients′ quality of life [5].

PCa can be curable if diagnosed when the development is still in its early stages [3]. For localized PCa, the gold standard treatment is radical prostatectomy (RP). However, around 40% of the patients will develop biochemical recurrence (BCR) after RP, which indicates PCa progression [10]. After RP, levels of PSA decrease until undetectable, and the resurgence of high PSA levels is the first indication of BCR. The ideal PSA cut-off to define BCR is still controversial [10], with the American Urological Association and the European Association of Urology defining BCR for a serum PSA ≥ 0.2 ng/mL [10,11]. For aggressive PCa, one of the most frequently used treatments is androgen deprivation therapy (castration). However, the treatment can be hampered by the development of resistance to castration [12]. 

Considering the limitations of the currently available PCa diagnostic tools, the scientific community has performed massive efforts to discover new biomarkers for PCa detection. These biomarkers include several derivatives of PSA, like the prostate health index (PHI) and the 4Kscore tests. PHI test combines the PSA precursor isoform that circulates uncomplexed [−2]proPSA (p2PSA), free PSA (fPSA) and total PSA, through the formula PHI = (p2PSA/fPSA) × √(tPSA) [13,14]. Higher levels of PHI are correlated with PCa [13,14] and this test obtained FDA approval for men with PSA between 2.5 and 10 ng/mL and negative DRE [2]. The 4Kscore test includes total PSA, fPSA, intact PSA (iPSA), and human glandular kallikrein (hK2), a protein similar to PSA [2]. Despite the promising results, 4Kscore test did not obtain FDA approval [2].

With the raising of “omics’’ technologies, other biomarkers for PCa detection have been proposed, such as prostate cancer antigen 3 (PCA3), which is a biomarker coming from transcriptomic methodologies. PCA3 gene encodes a noncoding RNA which is specific of prostate and is increased in urine of PCa patients collected after DRE. Despite the controversy around the ideal cut-off for the levels of this biomarker, this test obtained FDA approval for men with high PSA levels and/or positive DRE and/or previous negative PB [15,16]. Prostarix test, which is also performed in urine after DRE, was developed using metabolomic approaches and detects four amino acids [17], namely sarcosine, glycine, alanine, and glutamate [13,16]. This test has not yet obtained FDA approval [13], but it is commercially available and is recommended for men with persistent PSA increase and previously negative PB [13,17].

Despite such great efforts to discover new biomarkers for PCa detection and the promising perspectives, no biomarker has so far been able to replace PSA in clinical practice for PCa screening, highlighting the need to pursue research in this field. In this review, we explore the potentialities and challenges of metabolomics for PCa biomarker discovery. In addition, we update our earlier review [18] by presenting the most recent metabolomic studies performed in urine and tissues from PCa patients aimed at evaluating metabolic pathways perturbed in this disease and the altered metabolites as potential biomarkers for PCa detection. For this, a search was conducted in the PubMed database for articles published between January of 2015 and December 2020, using the keywords “metabolomics”, “prostate cancer”, “biomarker”, “urine”, or “tissue”. A total of 25 studies were included, of which 12 were performed in PCa tissue samples, 12 in PCa urine samples and one study included both matrices.

## 2. Metabolomic Approaches to Biomarker Discovery

Nicholson et al. (1999) defined metabonomics as “the quantitative measurement of the dynamic multiparametric metabolic response of living systems to pathophysiological stimuli or genetic modification” [19]. Nowadays, the term metabonomics is often used interchangeably with the terms metabolic phenotyping, metabolic profiling, or simply metabolomics in the context of the comprehensive analysis of all metabolites of a biological sample representative of an organism or cell. Metabolomics is the last “omic” platform in the “omics” cascade (genomics—transcriptomics—proteomics—metabolomics), and it focuses on the study of small molecules (<1500 Da) [20] in several complex matrices like serum, saliva, exhaled air, urine, tissue, among others [21]. When compared with other omics, metabolomics shows important advantages: (i) the dynamic feature of metabolome, once it modifies rapidly in response to changes in cell status, allowing a continuous evaluation of the cell state [22]; (ii) minor changes in gene expression or protein synthesis are translated into major alterations in metabolite levels [23]; (iii) the response of metabolome to pathophysiological alterations is much more sensitive than gene or protein response [24]; (iv) the alterations in metabolome are closely related with the observed phenotype; (v) the levels of several metabolites can simultaneously be measured, allowing to establish a pattern of alterations associated with an specific pathophysiological state [22]; (vi) allows to define patterns of disease progression [25].

Human metabolome comprises metabolites of low molecular weight from very different chemical families, such as amino acids, lipids, nucleotides, carbohydrates, organic acids, among others. They are present in a wide range of concentrations and have distinct physicochemical characteristics [26]. When a metabolomics study is designed, the selection of the analytical technique is a critical step, once this choice will restrict the metabolites detected and consequently the obtained results [27]. This selection needs to take into consideration the characteristics of the analytical technique like sensitivity, resolution, limits of detection of the instrumental technique [27], but also the characteristics of the samples and of the metabolites of interest, e.g., metabolite physicochemical properties and abundance [27,28].

Currently, the majority of the metabolic studies are performed using mass spectrometry (MS), frequently coupled with a separation technique like gas or liquid chromatography (GC–MS or LC–MS), and nuclear magnetic resonance spectroscopy (NMR) [26,29]. MS and NMR show several differences, including in the detected range of concentrations, namely, MS allows the detection of metabolites in concentrations ranging from picomolar (pM) to millimolar (mM) [30] and NMR from micromolar (µM) to millimolar (mM) [31]. Table 1 summarizes the advantages and limitations of the three analytical techniques (GC–MS, LC–MS and NMR), for metabolomic studies. As depicted in Table 1, none of these methods are able to cover the entire metabolome. For example, GC–MS is only suitable for the analysis of thermally stable compounds, such as volatile organic compounds (VOCs) [26]. In turn, LC–MS is used for profiling of compounds with medium and low polarities (reversed-phase LC) and polar compounds (hydrophilic-interaction LC), but the datasets generated are complex, spectrometer dependent, and require additional MS/MS experiments, as well as spiking with authentic standards, in order to perform metabolite annotation and identification [26,32,33]. NMR shows a lower sensitivity, which compromise the detection of low abundance metabolites. Importantly, due to NMR nondestructive nature, the samples can be recovered after analysis and used in complementary studies (e.g., MS analysis) to obtain a more comprehensive characterization of the metabolome [34]. Indeed, the combination of more than one analytical platform is desirable to allow a more comprehensive analysis of a sample metabolome [26,29].

Metabolomic studies can follow two distinct approaches, namely the untargeted or the targeted approach. In the first, the goal is to cover the maximum of the metabolome detecting as many metabolites as possible in a matrix, and is frequently denominated as hypothesis generation [23]. In the second, a single metabolite or a group of metabolites (e.g., metabolites from a specific metabolic pathway) are previously selected and all the study is designed to detect and quantify these metabolites. This approach can be applied to validate the results obtained through an untargeted approach and is called hypothesis-driven [23,25]. 

Regarding PCa metabolomic studies, two main goals are recognized: (i) the discovery of biomarkers with high sensitivity and specificity for PCa timely detection and (ii) to understand the metabolic basis of PCa pathogenesis identifying altered metabolic pathways in consequence of PCa development and progression [25]. Nevertheless, the potential application of metabolomic studies is not limited to these two main goals, once metabolomic studies can also be applied to study the effectiveness of treatments, as well as the mechanism of action of therapeutic drugs and the mechanism of drug resistance or contribute to achieve the goal of personalized medicine [38].

Over the years, several independent subareas emerged from metabolomics, like volatilomics, lipidomics, among others. Volatilomics is based on the analysis of VOCs, like aldehydes, ketones, alcohols, hydrocarbons, or aromatic compounds [39], that are produced by human body and released into breath, blood, sweat, urine, feces, or saliva [39,40]. All VOCs share some physicochemical characteristics, such as low molecular weight and low boiling point and/or elevate vapor pressure in normal conditions [41]. The interest to investigate VOCs as potential cancer biomarkers gained strength after the observation that dogs were able to “smell” urine or skin samples of cancer patients with high sensitivity and specificity, indicating that the composition of VOCs is different in cancer individuals [42,43,44]. VOCs are end products of human biological activity and their composition in biological samples can reflect pathological processes [40], alterations in normal biochemical pathways and/or a response to a damage or disease. Indeed, cancer development and progression can lead to the production of new VOCs and/or to change their concentration [41], making them suitable candidates to cancer biomarkers [39]. One of the greatest advantages of VOCs as biomarkers is the possibility to easily, inexpensively and quickly detect them in clinical point of care through the most recent technological developments in biological sensors (e.g., electronic noses (e-nose)) [39]. 

Lipidomics is the subarea of metabolomics focused on the qualitative and quantitative profile of the lipid species in biological samples [45]. The knowledge of lipid metabolism is crucial to understand cancer development and progression for several reasons: (i) de novo synthesis provide phospholipids for cancer cell proliferation, (ii) fatty acid β-oxidation is important in energetics and redox homeostasis, (iii) lipids play an important role in signaling pathways [46] and, finally, (iv) lipids are extremely dynamic and can reflect physiological, pathological, and environmental alterations [47]. For these reasons, the interest to study the lipid profile of cancer cells has increased in the last years. It is estimated that mammalian cells comprise around 10,000 individual lipid species [48]. These lipids can be classified into different classes: (i) fatty acids, (ii) glycerophospholipids (GPLs), (iii) glycerolipids (e.g., triglycerides (TG)), (iv) saccharolipids, (v) sphingolipids (SL), and (vi) sterols. Each class of lipids show different biological functions. For instance, TG are important for energy storage, while sterols are key elements in cellular membrane and have also hormonal functions [49]. GPLs and SL are important components of cellular membranes and lysophospholipids (LPLs) (a subclass of GPLs) are important molecules for cellular signaling. These three classes (GPLs, SL and LPLs) are the most frequently studied in cancer lipidomic studies. GPLs can still be divided into phosphatidylcholine, phosphatidylethanolamine (major components of human cellular membranes), phosphatidic acid, phosphatidylglycerol, phosphatidylinositol and phosphatidylserine, considering the molecular structure of these molecules. SLs can also be divided into several subclasses like ceramides, sphingomyelins, among others [45]. This summary reflects the importance and the complexity of the lipidome and justify that lipidomics comprises an independent subarea of metabolomics. Furthermore, several studies revealed that cancer cells show alterations in lipidome fingerprint demonstrating the potential of lipids as biomarkers and/or therapeutic targets [46,49]. 

## 3. The Metabolic Phenotype of Prostate Cancer

It is well established that cancer cells suffer profound metabolic alterations that are indispensable for cancer development and progression [50]. One of the most well described metabolic alterations of cancer cells is the Warburg effect, which is characterized by a change in the preferential pathway to produce energy. Indeed, cancer cells preferentially produce ATP via aerobic glycolysis, even in the presence of oxygen, while normal cells produce ATP through oxidative phosphorylation [50,51]. This shift leads to an increase in glucose uptake and in lactate secretion [50,52]. The increase in lactate levels seems to play an important role in cancer development and progression [50]. Lactate can be utilized as fuel for oxidative metabolism, metabolized into alanine and glutamine and can also intervene in cancer cell mobility, immune escape and angiogenesis [50].

**Figure 1:**
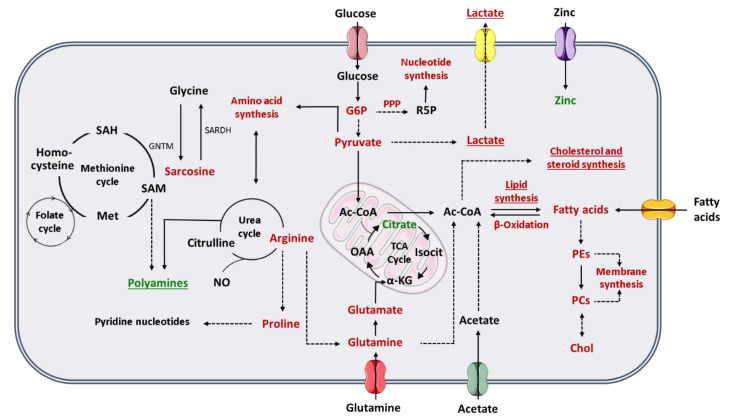
Schematic representation of the metabolic phenotype of prostate cancer cells. Red indicates increase in either metabolites or metabolic pathway flux and green indicates decrease in either metabolites or metabolic pathway flux. Underline indicates changes especially important in advanced PCa. The dashed lines represent multiple steps reactions. (α-KG, alpha-ketoglutarate; Ac-CoA, acetyl-coenzyme A; Chol, choline; G6P, glucose-6-phosphate; GNMT, glycine N-methyltransferase; Isocit, isocitrate; Met, methionine; NO, nitric oxide; OAA, oxaloacetate; PCs, phosphatidylcholines; PEs, phosphatidylethanolamines; PPP, pentose phosphate pathway; R5P, ribose-5-phosphate; SAH, S-adenosylhomocysteine; SAM, S-adenosylmethionine; SARDH, sarcosine dehydrogenase; TCA cycle, tricarboxylic acid cycle).

To comprehend how the Warburg effect impacts PCa cell metabolism, it is important to revisit the peculiar metabolic phenotype of normal prostate cells. Contrarily to other human cells, prostate cells favor citrate accumulation instead of citrate oxidation for energy production through tricarboxylic (TCA) cycle, also known as Krebs cycle or citric acid cycle [53]. Prostate cells have an increase in the zinc transporter ZIP1 and, consequently, zinc accumulates in prostate tissue [52]. The high levels of zinc are responsible for the inhibition of m-aconitase, which is the enzyme responsible for citrate oxidation in TCA cycle [53]. However, one of the first metabolic alterations associated with PCa development is the loss of cell ability to accumulate zinc and subsequent reduction of citrate levels in PCa cells [53]. Indeed, there is an increment of citrate oxidation in TCA cycle to produce energy in PCa cells [52,53]. For this reason, the Warburg effect and consequent increase in aerobic glycolysis is described mainly in advanced stages of PCa, where the increase in glycolytic pathway is associated with metastases formation and thereafter to a poor prognosis [52,54]. Furthermore, citrate can also be used in PCa cells to produce acetyl-coenzyme A (acetyl-CoA) (important for fatty acids and cholesterol synthesis) and oxaloacetate (amino acid precursor) [55] (Figure 1). Beyond citrate accumulation, normal prostate cells can also accumulate polyamines, such as spermine and spermidine once they are important components of prostatic secretions [56]. Polyamine levels also decrease, similarly to citrate, during cancer development and progression (Figure 1). Indeed, this reduction in polyamine levels may promote PCa cell survival by preventing apoptosis [55,56].

Pentose phosphate pathway (PPP) is also altered in PCa cells, once the levels of glucose-6-phosphate dehydrogenase (a key enzyme in PPP) are increased through androgen receptor (AR) signaling [54], which is essential for PCa progression. AR signaling also promotes glycolysis and anabolism [55]. As previously referred, one of the most frequently used treatments for aggressive PCa is androgen deprivation therapy, which is associated to the development of castration-resistant state and consequently alterations in the lipid profile, and to a worse prognosis [55,57]. Furthermore, PCa cells show the ability to synthesize sterols, highlighting the importance of androgen signaling in PCa [57] (Figure 1).

Alterations in different amino acids, such as glutamine, have been associated with PCa and other cancers [50,52]. Glutamine is one of the most abundant amino acids in human plasma and has important roles in human metabolism [54], as it can be converted in glutamate, and subsequently be transformed in α-ketoglutarate, an intermediate in TCA cycle [50,52]. This amino acid can also be used by cancer cells for acetyl-CoA production [54], for fatty acid synthesis [52] and as a nitrogen and carbon donor for nucleotide, lipids and protein synthesis [50,54]. The glutamate resulting from glutamine is an essential substrate for glutathione synthesis, and therefore important for the protection of the cells against oxidative damage [50] (Figure 1). Arginine is an important amino acid involved in PCa metabolism. Arginine is converted by PCa cells in glutamine and/or proline [52]. The increase in proline levels is needed to the maintenance of the levels of pyridine nucleotides [54]. Arginine has also an important role in nitric oxide (NO) production [52] (Figure 1). 

Sreekumar et al. (2009), reported higher levels of sarcosine in urine of PCa patients, which was a milestone in PCa metabolomics [58], but its importance as potential PCa biomarker was refuted in the following years [59,60,61]. Sarcosine is synthesized from other amino acids, glycine, and vice versa. This reaction can be linked to methionine cycle, and the produced methionine can be up-taken to folate cycle. The combination of these two cycles is referred frequently as one-carbon metabolism. One-carbon metabolism fuels building blocks for purines and thymidylates synthesis, which are essential for DNA synthesis and repair [62,63]. Methionine cycle also plays a role in polyamines and glutathione synthesis [54,63] (Figure 1). 

Another prominent characteristic of a cancer cell is its ability to proliferate constantly. Lipids are major components of cellular membranes, so alterations in lipids, and in choline or choline derivative metabolites, have a very important role in cancer cells proliferation [53,54]. Furthermore, lipids are also essential as energy resource, for energy storage and for intracellular signaling [54]. Therefore, increase in *de novo* fatty acids synthesis is an initial event in PCa development, which is stimulated by androgen signaling [52], as well as the increase in fatty acids oxidation to produce energy [52,54]. The importance of lipogenesis in PCa is patent in the increase of the expression of lipogenic and lipid-modifying enzymes, occurring in PCa [52,54] and by the accumulation of triglycerides, cholesterol esters and phospholipids (phosphatidylcholine), mainly in aggressive PCa [53]. Furthermore, metastatic PCa cells also show an upregulation of acetyl-CoA synthetase 2, allowing PCa cells to produce acetyl-CoA (essential for fatty acids synthesis) from acetate, while normal cells produce acetyl-CoA essentially from glucose and glutamine [54] (Figure 1). Moreover, PCa cells show the ability to take up exogenous lipids and to synthetize and mobilize lipids storage in other cells, like adipocytes [54]. 

From this brief explanation, it is reasonable to infer that the study of the metabolic signature of cancer cells has an enormous potential in the discovery of new biomarkers, as well as to elucidate cancer pathophysiological mechanisms, which can be used to define new therapeutic strategies.

## 4. Tissue Metabolomic Studies

The collection of tissue samples is very invasive, hampering their use for PCa scree-ning. However, the study of the tissue metabolome has important advantages, once this is the ideal matrix to establish which metabolic alterations are specific to PCa development and progression. Furthermore, tissue studies have been performed using matched tumoral and nontumoral samples from the same individual, thus minimizing the contribution of confounding factors (e.g., age, comorbidities, lifestyle).

**Figure 2:**
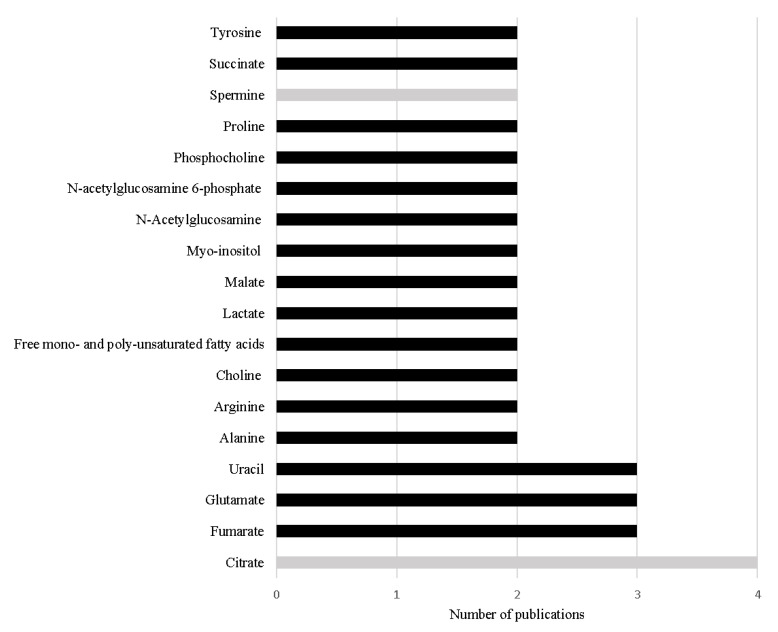
Metabolites referred with the same variation in more than one study performed in PCa tissue in the last 5 years. The black bars represent metabolites increased in PCa and the grey bars represent metabolites decreased in PCa.

Thirteen metabolomic studies performed in PCa tissue samples were published in the last 5 years, including two lipidomic studies. Table 2 summarizes the study design and main outcomes obtained in those studies. Overall, a total of 98 different metabolites were associated with PCa, indicating that PCa is related with dysregulations in 32 different metabolic pathways (Table 2). Interestingly, 18 metabolites were found to be common among the included studies (Figure 2). It is important to note that these studies were performed under different analytical conditions, with different sample selection criteria and using different statistical approaches, foreseeing difficulties to compare results across studies. The fact that these metabolites were found common in the various studies, highlights their importance in PCa metabolism and their potential as specific PCa biomarkers.

The most frequent alteration reported among the studies conducted in the last 5 years is the significant decrease in citrate levels in PCa tissue [68,74,75,76] (Table 2 and Figure 2). This result is not unexpected as the loss of capability to accumulate citrate is one of the first metabolic alterations observed in prostate cells during malignant transformation. This loss of capability to accumulate citrate translates in a profound alteration in energetic metabolism of PCa cells, once PCa cells start to use citrate in TCA cycle more efficiently than normal prostate cells [51,52]. Furthermore, Braadland et al. (2017) compared PCa tissue from men that suffered PCa recurrence after prostatectomy with tissue from men that, until the date of the study, did not show signals of recurrence, unveiling that lower levels of citrate in PCa tissue were associated with shorter time of recurrence [70]. Additionally, lower levels of citrate were also associated with more aggressive PCa [77]. Beyond citrate, two other metabolites involved in energetic metabolism, namely alanine [69,71] and lactate [69,72] showed significant alteration in PCa tissue.

The increased levels of other key metabolites of TCA cycle have also been frequently cited in the reviewed studies, namely succinate [71,76], malate [71,76] and fumarate [71,74,76] (Table 2, Figure 2). The increase in malate and fumarate levels was also correlated with Gleason score [71,78] and tumor stage [71]. Notably, both succinate and fumarate were previously considered oncometabolites [49], once their accumulation leads to cancer progression [79]. The increased levels of succinate and fumarate have been asso-ciated in other cancer types (e.g., paraganglioma, pheochromocytoma or kidney cancers [78]) with mutation in the enzymes succinate dehydrogenase (SDH) and fumarate hydratase (FH), respectively [49,80]. However, these results were not observed in PCa studies performed by Shao et al. (2018), which suggested the involvement of other mechanisms that could also be related with the increase of the levels of these metabolites in PCa [71]. Once fumarate is also linked with urea cycle [74,76], this metabolic pathway could be res-ponsible for keeping the high levels of fumarate in PCa tissue [74,79]. As previously referred, the accumulation of fumarate leads to cancer progression, this could involve the activation of hypoxia-inducible factor 1-subunit alfa (HIF1α) and NFκB pathways [74]. HIF1α plays an important oncogenic role in PCa once this pathway is responsible for many essential mechanisms to guarantee PCa cell survival, like antiapoptosis, angioge-nesis and increased glycolytic metabolism. Furthermore, HIF1α protects PCa cells against oxidative stress and against the cytotoxicity caused by androgen deprivation therapy, chemotherapy, or radiation [81]. Similarly, NFκB pathways support PCa cell survival, proliferation, and invasion, playing an important role in the development of resistance to castration therapy [82].

The increase in uracil levels is another alteration consistently reported in PCa tissue (Table 2, Figure 2), suggesting that PCa cells have alterations in pyrimidine metabolism [65,71,76]. Pyrimidine metabolism is a complex biochemical pathway that comprises diffe-rent reactions, namely de novo nucleotide synthesis, nucleoside salvage, and pyrimidines degradation [83]. Pyrimidines, like uracil [84], are essential in cells metabolism once they are constituents of nucleotides, nucleic acids, vitamins, proteins, and folates [85]. Furthermore, they are key intermediates in RNA and DNA synthesis, protein and lipids glycosylation, synthesis of phospholipid precursors [84,85], and in reactions of glucuronidation [84]. Cancer cells are dependent on de novo nucleotide synthesis for cell proliferation and consequently for cancer development and progression [83,84]. Importantly, the inhibition of this metabolic pathway is a strategy adopted in the treatment of several cancers (e.g., colorectal cancer and pancreatic cancer) [84,86,87]. 

One of the main functions of normal prostate is to synthesize polyamines like spermine but apparently this function is impaired with PCa development and progression [70] leading to a decrease in the levels of spermine [68,76]. Indeed, the reduction of spermine levels was proposed as a biomarker able to predict BCR [70]. Interestingly, levels of spermidine, a spermine precursor, were also reported as significantly altered in PCa tissue samples; however, the obtained results were contradictory. Huan et al. (2016) found a significant increase in the levels of spermidine [65], whereas Dudka et al. (2020) showed a significant decrease in the levels of this metabolite in PCa tissue samples [76]. 

As previously referred, lipid metabolism can be an important source of PCa biomarkers, emphasizing the relevance of lipidomic studies. The major reported lipidic alterations occurring in PCa cells involved phospholipids from cellular membrane [64,66,67,69,71,72,73,76] which was expected taking into consideration that cancer cells show a high proliferative phenotype. Notably, the significant decrease in the levels of LPC (16:0) was able to predict BCR [64]. This observation is supported by a transcriptomic study, that evaluates the expression of the enzyme LPC transferase 1 (LPCAT1). The increase in the expression of this enzyme was able to discriminate PCa from benign tissue, as well as to differentiate PCa with different GS and to predict BCR and/or metastasis development [88]. Furthermore, phosphocholine also revealed to be able to discriminate PCa tissue with different GS [76].

Finally, alterations in amino acid metabolism have also been widely reported in PCa, mainly in the increase levels of glutamate [72,75,76], tyrosine [74,75], arginine [74,76], and proline [71,74]. Importantly, the significant alteration in the levels of the first three amino acids was associated with GS and consequently PCa aggressiveness [76], making these metabolites potential diagnosis and prognosis biomarkers.

## 5. Urine Metabolomic Studies

Urine is the ideal matrix to be used in a screening test, due to its noninvasive nature, along with ease of collection and handling, high volume which allow repeated analysis, and lower complexity when compared with other biofluids (e.g., serum or plasma) [20,37,89]. Furthermore, urinary metabolites are concentrated by the kidneys, which are anatomically close to the prostate [89,90]. However, urine composition can vary due to several external factors, like diet, smoking habits, genetic factors, microbiota, diurnal cycles diabetes, and other diseases which can affect urine metabolome [91].

From 2015 to 2020, 13 studies performed in PCa urine samples were published, including four volatilomics studies. Table 3 summarizes these studies, highlighting study design, altered metabolites, metabolic pathways, as well as candidate biomarkers, whenever available. Overall, 179 different metabolites were associated with PCa, indicating that PCa is correlated with dysregulations in 48 different metabolic pathways. In this section, the metabolites that hold greatest potential as PCa biomarkers will be highlighted, considering different selection criteria: (i) consistency among different urinary studies, (ii) AUC greater than PSA and (iii) translatability between tissue and urine studies.

**Figure 3:**
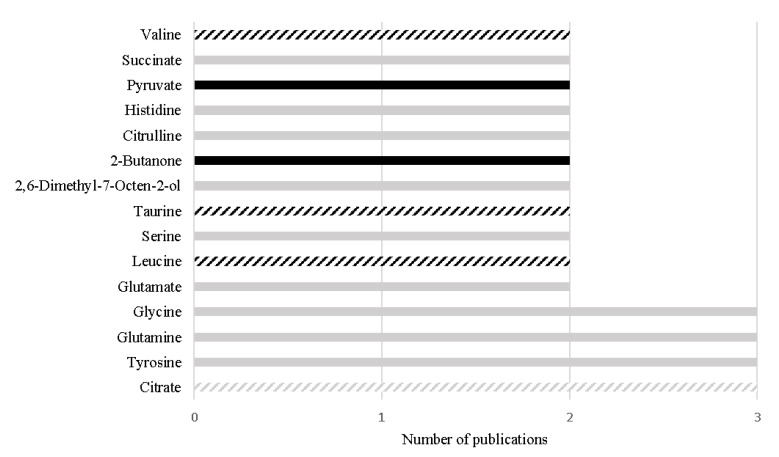
Metabolites found with the same alteration in urine metabolome of PCa patients in more than one study, in the last 5 years. The black bars represent metabolites increased in PCa and the grey bars represent metabolites decreased in PCa. The listed bars correspond to the metabolites that were previously found with the same variation in PCa tissue.

Despite the great differences (e.g., different analytical platform, samples preparation or different inclusion/exclusion criteria) among the study designs, 15 metabolites have been consistently reported with the same variation among different urinary studies, as represented in Figure 3. Importantly, four metabolites out of the 15 have also been reported with the same alteration in PCa tissue, namely decreased levels of citrate [68,74,75,76,92,100], increased levels of leucine [74,97,102], increased levels of valine [74,97,102], and increased levels of taurine [96,100,104], suggesting that these alterations may be specific of PCa tumors and suggesting their translatability between tissue and urine samples (Figure 3). 

In addition, 12 metabolites stood out once they unveiled similar or even better performance than PSA (AUC ranging from 0.53 to 0.83) for PCa detection [7], namely γ-amino-n-butyric acid (AUC: 0.93), phosphoethanolamine (AUC: 0.88), ethanolamine (AUC: 0.86), homocitrulline (AUC: 0.84), asparagine (AUC: 0.773), arginine (AUC: 0.83) [96], spermine (AUC: 0.83) [94], δ-hydroxylysine (AUC: 0.80) [96], 2-hydroxyvalerate (AUC: 0.76), 2-furoylglycine (AUC: 0.74), mannitol (AUC: 0.69), and glucose (AUC 0.69) [102]. From these 12 metabolites, the alterations observed in the levels of three metabolites were previously reported in PCa tissues, namely spermine [68,76], ethanolamine [105] and glucose [76]. Importantly, the decrease in glucose levels was also correlated with GS [76]. The significant alterations observed in the other nine metabolites, to the best of our knowledge, were not previously reported in PCa tissue. It is important to highlight that even if it is not possible to prove translatability of a metabolite from tissue to urine, this does not invalidate its potential as PCa biomarker once its alteration can for example be driven from a systemic response to PCa development and progression.

The volatilomic studies have been more focused in the definition of biomarker panels for possible detection through biosensors rather than proposing individual biomarkers [93,99,103]. The smallest biomarker panel reported included four metabolites (2,6-dimethyl-7-octen-2-ol, 3-octanone, 2-octanone and pentanal) [93], which unveiled accuracies at least equal to PSA (accu. of 62–75% for PSA vs. accu. of 63–65% for the 4-biomarker panel [7,93]. Remarkably, a 6-biomarker panel (hexanal, 2,5-dimethylbenzaldehyde, 4-methylhexan-3-one, dihydroedulan IA, methylglyoxal, 3-phenylpropionaldehyde) [99] and an improved 10-biomarker panel (methylglyoxal, hexanal, 3-phenylpropionaldehyde, 4-methylhexan-3-one, 2.5-dimethylbenzaldehyde, dihydroedulan IA, ethylbenzene, heptan-2-one, heptan-3-one, 4-(2-methylpropoxy)butan-2-one, methyl benzoate, 3-Methylbenzaldehyde) [103] were recently proposed that outperformed PSA in all performance parameters (PSA: acc = 62–75%, sens = 20.5%, spec = 51–91% [4,7,8]; 6-biomarker panel: acc = 86%, sens = 89%, spec = 83% [99]; 10-biomarker panel: acc = 92%, sens = 76%, spec = 90% [103]). Notably, the 10-biomarker panel proved to be able to differentiate PCa from cancer-free individuals as well as from other urological cancers (renal and bladder cancers) [103].

Overall, findings from the reviewed studies showed that PCa development and progression is mainly associated with alterations in amino acid metabolism, energy metabolism, especially in TCA cycle, and membrane metabolism (Table 2 and Table 3).

## 6. Current Challenges and Future Perspectives

There are no doubts that the scientific community has made enormous efforts to define the impact of PCa in human metabolome with dozens of studies focused on this topic, not only using tissue and urine matrices but also other biological samples as serum, plasma, seminal fluid, prostatic fluid, and even cell lines [18]. However, some biological and technical challenges should be addressed before we can translate all the potentialities of metabolomics into clinical practice.

The traditional paradigm is to find a single biomarker for PCa screening. However, during the last years, the idea of using a panel of biomarkers instead of a single biomarker has gained strength, especially in volatilomic studies. The use of a biomarker panel has important advantages, once a multi-biomarker panel may be able to capture more deeply the various metabolic dysregulations occurring during cancer development and progression than a single biomarker [106]. Hence, a multi-biomarker panel allows the definition of a more robust signature of PCa providing a better evaluation of cancer progression. Furthermore, the use of a biomarker panel avoids that an arbitrary change in a single metabolite leads to a false result [22].

As referred in the previous section, the comparison of the findings from different studies is compromised by the lack of standardized procedures in metabolomic studies, especially in study design which consequently increases interlaboratory variability [107]. Many efforts have been made to accomplish the goal of standardized procedures in metabolomics studies in the last years [107,108]. There is still a long way to go until the desired standardization, but the first steps have already been taken [108].

Other crucial technical challenge is metabolite identification. This is particularly true in volatilomics studies. Additionally, the interpretation of the urinary volatilome signature of PCa is particularly challenging once there is no clear understanding of the biological origin of VOCs [39]. In addition, the volatilome of PCa tissue has not been explored so far, to our knowledge, hindering the elucidation of a potential translatability of VOCs from tissues to urine samples. In future, this issue can be addressed through volatilomics studies of PCa tissue and fluxomic studies. Fluxomic studies allow the understanding of the metabolic origin of endogenous VOCs by labeling and tracking metabolic precursors (e.g., glucose), throughout the metabolic pathways [109].

Perhaps the greatest limitation to biomarker discovery relies on the fact that several metabolomic studies are essentially descriptive and skip the validation step. Indeed, the vast majority of the papers just list which metabolites are statistically different between the groups in study, not proposing candidate biomarkers and/or clearly state the performance of the proposed metabolites/biomarkers (e.g., AUC, sensitivity, specificity and/or accuracy), thus impairing the discussion of which would be the most promising biomar-kers for PCa. Hence, future studies should be less descriptive and more assertive, propo-sing and evaluating potential biomarkers. Furthermore, it is also important to include external sets for model/results validation to improve the robustness of candidate biomarkers and to include unambiguous biochemical and biological interpretation of PCa metabolic dysregulations. Remarkably, some of the studies included in this revision are already fo-llowing this direction. In addition, it is well known that especially urinary metabolic profile can be affected by several factors (e.g., diet, lifestyle, microbiota, race, among others) [107], so it is also crucial to perform studies in large and more heterogeneous populations (e.g., American, African, Caucasian), to ensure that the proposed biomarkers can be applied among different countries and different lifestyles.

To conclude, metabolomics is a powerful tool to uncover the metabolic signature of PCa development and progression. The results obtained so far in tissue and urine metabolomic studies unveiled potential to define new screening/diagnosis biomarkers.

## Figures and Tables

**Table 1:** Main advantages and limitations of gas or liquid chromatography coupled with mass spectrometry (GC–MS or LC–MS), and nuclear magnetic resonance spectroscopy (NMR) in metabolomic studies.

Analytical Platform	Advantages	Limitations
GC–MS	- Ideal for volatile organic compounds detection [26] - High sensitivity and resolution [26] - Available database for metabolite identification [26] - High peak capacity to cover a wide range of concentrations [22] - Small amounts of sample used [32] - High dynamic range, selectivity and throughput [26,29,35] - Retention times are highly reproducible [22,26]	- Only suitable for thermally stable compounds [26] - Derivatization step is required for nonvolatile compounds [26] - Formation of new compounds due the derivatization step [28] - Destructive nature [32]
LC–MS	- Detects a wide range of metabolites, including conjugates, of varying molecular weight and different natures (hydrophilic and hydrophobic compounds) [22,26] - Easy sample preparation [26,28] - Does not require derivatization [22] - Small amounts of sample used [32]	- Destructive nature [32] - MS/MS experiments are usually required for metabolite identification, which implies additional experimental time [33]
NMR	- Relatively high throughput and efficiency [22,36] - High reproducibility and selectivity [34,37] - Nondestructive nature [22,34] - Analysis of liquid and solid matrices [34] - Easy sample preparation [37] - Provides information about chemical structure, chemical environment and molecular interactions [34,36]	- Low sensitivity [34,37] - High costs [22] - Not optimal for targeted analysis [37] - Peak overlapping which difficult quantification [34]

**Table 2:** Metabolomic studies performed in tissue samples from PCa patients in the last 5 years (2015–2020).

PCa Group	Control Group	Analytical Platform	Statistical Methods	Altered Metabolites (Direction of Variation)	Dysregulated Metabolic Pathways	Candidate Biomarkers	Ref.
*n* = 31	*n* = 14 (benign adjacent tissue)	HR-MALDI-IMS MS/MS	Univariate and Multivariate Cox Regression Analyses	1. LPC (16:0) (−) 2. SM [(d18:1/16:0)] (−) Predictor of biochemical recurrence: 1. LPC (16:0) (−)	1. FAs de novo synthesis and remodeling pathway (Lands′ pathway) 2. Arachidonic acid metabolism	LPC (16:0)	[64]
*n* = 25 Validation set 1: *n* = 19 Validation set 2: *n* = 12	*n* = 25 (normal adjacent tissue) Validation set 1: *n* = 17 (normal adjacent tissue) Validation set 2: *n* = 12 (normal adjacent tissue)	LC–MS	PCA OPLS-DA Model performance: Sens: 85% Spec: 83–91% AUC: 0.90	1. Adenosine monophosphate (−) 2. Spermidine (+) 3. Uracil (+)	1. Purine metabolism 2. Polyamines synthesis 3. Pyrimidine metabolism	Adenosine monophosphate (AUC: 0.82) Spermidine (AUC: 0.85) Uracil (AUC: 0.91)	[65]
*n* = 25 Validation set: *n* = 51	*n* = 25 (normal adjacent tissue) Validation set: *n* = 19 (BPH)	LC–MS	PCA PLS-DA Model performance: AUC: 0.90–0.94 External validation: AUC: 0.84–0.91	1. PCs (alkyl/acyl-PCs, PC-O) (−); PEs (alkenyl/acyl-PEs, plasmalogens, PE-P) (−); Free saturated FAs (−); Diacyl-PC (+); Diacyl-PE (+); Free mono- and poly-unsaturated FAs (+) 2. CEs (+); Cholesteryl oleate (+)	1. Lipogenesis, lipid uptake and phospholipids remodeling 2. Cholesterol metabolism	Cholesteryl oleate (AUC: 0.91(PCa vs. normal adjacent tissue) and AUC: 0.96 (PCa vs. BPH))	[66]
*n* = 25 Validation set: *n* = 51	*n* = 25 (normal adjacent tissue) Validation set: *n* = 51 (benign adjacent tissue) + *n* = 16 (BPH)	LC–MS	PCA PLS-DA	1. Choline (+); Citicoline (+) Nicotinamide adenine dinucleotide (+); S-Adenosylhomoserine (+); 5- Methylthioadensine (+); S-Adenosylmethionine (+); Nicotinamide mononucleotide (+); Nicotinamide adenine dinucleotide phosphate (+); Adenosine (−); Uric acid (−) 2. D-Glucosamine 6-phosphate (+); N-Acetyl-D-glucosamine (+); N-Acetyl-D-glucosamine 6-phosphate (+); UDP-Acetyl-glucosamine (+) 3. 2-Aminoadipic acid (+); Saccharopine (+); Trimethyllysine (+); Carnitine C4-OH (+); Carnitine C14:2 4. Sphingosine (+) 5. Pantothenic acid (+) 6. Dehydroepiandrosterone sulfate (−); Etiocholanolone sulfate (−) 7. Phenylacetylglutamine (−)	1. Cysteine and methionine metabolism; NAD metabolism; phospholipid membrane metabolism 2. Hexosamine biosynthesis 3. Lysine degradation; β-oxidation of FAs 4. Sphingolipid metabolism 5. CoA homeostasis 6. Dihydro-testosterone synthesis 7. Unavailable	Sphingosine (AUC: 0.81–0.87)	[67]
*n* = 34 (ERG_high_ PCa)	*n* = 30 (ERG_low_ PCa)	HR-MAS ^1^H-NMR	PCA PLS-DA Model performance: Sens: 79% Spec: 74% Accu: 77%	ERG_high_ PCa vs. ERG_low_ PCa 1. Citrate (−) 2. Spermine (−)	1. TCA cycle 2. Polyamines synthesis	Citrate and spermine ERG_high_ for stratification	[68]
*n* = 6 (patients treated with Degarelix) + *n* = 7 (untreated)	*n* = 10 (benign from untreated patients)	HR-MAS ^1^H-NMR	PCA OPLS-DA	Untreated patients: 1. Lactate (+); Alanine (+) 2. Total choline (+) Patients treated with Degarelix: 1. Lactate (−) 2. Total choline (−)	1. Energetic metabolism 2. Choline metabolism; Phospholipid membrane metabolism	Lactate Total choline	[69]
*n* = 50 (patients that developed recurrence after prostatectomy)	*n* = 60 (patients that did not develop recurrence after prostatectomy)	HR-MAS ^1^H-NMR	PLS-DA Model performance: Sens: 92% Spec: 92% Accu: 92%	Increased risk of recurrence 1. (Total choline + creatine)/spermine (+); (Total choline + creatine)/citrate (+) 2. Spermine (−) 3. Citrate (−)	1. Choline metabolism; Phospholipid membrane metabolism 2. Polyamines synthesis 3. TCA cycle	Spermine Total choline + creatine/spermine	[70]
*n* = 21 Validation set: *n* = 50	*n* = 21 (benign adjacent tissue) Validation set: *n* = 50	GC–MS	OSC-PLS-DA	1. Fumarate (+); Malate (+); Succinate (+); 2- Hydroxyglutaric acid (+); Alanine (+); Glycerol-3-phosphate (+) 2. 11-Eicosenoic acid (+); Docosanoic acid (+); Eicosanoic acid (+) 3. Glycerolipids (+); *Myo*-inositol (+) 4. Uracil (+) 5. Proline (+)	1. Energetic metabolism (TCA cycle) 2. FAs metabolism 3. Membrane metabolism 4. Pyrimidine metabolism 5. Amino acid metabolism	-	[71]
*n* = 199 Validation set *n* = 166	*n* = 179 (benign adjacent tissue) *n* = 15 (BPH) + *n* = 14 (cancer-free patients) Validation set *n* = 159 (benign adjacent tissue)	HR-MAS ^1^H-NMR	Linear Regressions	1. *Myo*-inositol (+); Phosphocholine (+); Glycerophosphocholine (+) 2. Lactate (+); Taurine (−) 3. Histidine (+) 4. Phenylalanine (−); Glutamate (+)	1. Membrane metabolism 2. Energetic metabolismo 3. Histidine metabolism 4. Amino acid metabolism	*Myo*-inositol	[72]
*n* = 13 (American African population) + *n* = 13 (Caucasian American population)	*n* = 12 (American African population) + *n* = 9 (Caucasian American population) (benign adjacent tissue)	GC-FID ESI–MS	Generalized linear model	Saturated total FAs (+); Arachidic acid (+); Myristic acid (+) Monounsaturated total FAs (+); Polyunsaturated FAs (+); n-6 Total FAs (+); n-3 Free FAs (+)	Lipid metabolism	Arachidic acid (sens: 78%; spec: 75%; accu: 80%) (American African population) Myristic acid (sens: 85%; spec: 89%; accu: 98%) (Caucasian American population)	[73]
*n* = 13	*n* = 13 (benign adjacent tissue)	LC–MS CE–MS	OPLS-DA	1. Cysteine (+); Lysine (+); Methionine (+); Phenylalanine (+); Tyrosine (+); Branched-chain amino acids (leucine, isoleucine, and valine) (+); Fumarate (+) 2. Glycerophospholipids (+) 3. Fructose 6-phosphate (−); Fructose 1,2-biphosphate (−); Pyruvate (−); Citrate (−); *cis*-Aconitate (−); Isocitrate (−) 4. N-Acetylglucosamine (+); N-Acetylglucosamine 1-phosphate (+), N-acetylglucosamine 6-phosphate (+); Galacturonate 1-phosphate (+) 5. Aspartate (+); Argininosuccinate (+); Arginine (+); Proline (+); Fumarate (+)	1. Amino acid metabolism 2. Lipid metabolism 3. TCA cycle 4. Hexosamine pathway 5. Urea cycle	Fumarate Citrate Isocitrate	[74]
*n* = 58	*n* = 18 (BPH)	^1^H-NMR	PCA PLS-DA	1. Creatine (−); Creatinine (−); Glutamate (+); Glutamine (+); Formate (+); Tyrosine (+); Uridine (+) 2. Citrate (−) 3. Trimethylamine (+)	1. Amino acid metabolism 2. TCA cycle 3. Membrane metabolism	Citrate Glutamine	[75]
*n* = 70 43 GS (3 + 3) 16 GS (3 + 4) 10 GS (4 + 3) 1 GS (4 + 4)	*n* = 59 (benign adjacent tissue)	^1^H HR MAS NMR ^1^H/^31^P NMR LC–MS	PCA OPLS-DA	PCa vs. Benign 1. Citrate (−); Succinate/ malate (+); Fumarate (+) 2. Putrescine (−); Spermidine (−); Spermine (−) 3. Glutamate (+) 4. Uracil (+) 5. Hypoxanthine (+); Inosine (+) 6. α-Glucose (−) 7. SM (−) 8. NAD^+^ (−) 9. Phosphocholine (+); PE (+); LPC (−); 10. Arginine (+); 11. Docosapentanoic acid (22:5) (+); Oleic acid (18:1) (+); Linoleic acid (+); Docosahexaenoic acid (22:6) (+); Maleic acid (+); GS ≥7 vs. GS 6 3. Glutamate (+) 5. Hypoxanthine (+) 6. α-Glucose (−) 7. Sphingosine (+) 9. Glycerophosphorylcholine (+); Phosphocholine (+) 10. Arginine (+) 11. Hexanoylcarnitine (+) 12. Tyrosine (+); Valine (+); Phenylalanine (+) 13. Ascorbate (+) 14. 2-Hydroxybutyrate (+) 15. Lysine (+); Threonine (+)	1. TCA cycle 2. Polyamines synthesis 3. Glutamate metabolism 4. Pyrimidine metabolism 5. Purine metabolism 6. Glycolysis 7. Sphingolipid metabolism 8. Nicotinate and nicotinamide metabolism 9. Glycerophosphocholine metabolism; Phospholipid membrane metabolism 10. Urea cycle 11. Free FAs oxidation 12. Branched-chain amino acid meta-bolism 13. Inositol metabolism 14. Propanoate metabolism 15. Aminoacyl-tRNA biosynthesis	Phosphocholine Glutamate Hypoxanthine Arginine α-Glucose	[76]

Notes: (+) indicates increased levels in PCa, (−) indicates decreased levels in PCa; the numbering of the column *Altered Metabolites* is related with the numbering of the column *Dysregulated metabolic pathways*. Abbreviations: ^1^H-NMR, proton nuclear magnetic resonance spectroscopy; ^31^P NMR, phosphorus-31 nuclear magnetic resonance spectroscopy; accu, accuracy; AUC, area under the curve; BPH, benign prostatic hyperplasia; CE–MS, capillary electrophoresis–mass spectrometry; CEs, cholesteryl esters; PCs, ether-linked phosphatidylcholines; ESI–MS, electrospray ionization–mass spectrometry; ERG, ETS-related gene; FAs, fatty acids; GC-FID, gas chromatography-flame ionization detector; GC–MS, gas chromatography–mass spectrometry; GS, Gleason score; HR-MALDI-IMS, high-resolution matrix-assisted laser desorption/ionization imaging mass spectrometry; HR-MAS ^1^H-NMR, high resolution magic angle spinning proton nuclear magnetic resonance; LC–MS, liquid chromatography–mass spectrometry; LPC, lysophosphatidylcholine; OPLS-DA, orthogonal projections to latent structures discriminant analysis; OSC-PLS-DA, orthogonal signal corrected partial least squares-discriminant analysis; PCA, principal component analysis; PEs, phosphatidylethanolamines; PLS-DA, partial least squares-discriminant analysis; sens, sensitivity; spec, specificity; SM, sphingomyelin; TCA, tricarboxylic acid cycle.

**Table 3:** Metabolomic studies performed in urine samples from PCa patients in the last 5 years (2015–2020).

PCa Group	Control Group	Analytical Platform	Statistical Methods	Altered Metabolites (Direction of Variation)	Dysregulated Metabolic Pathways	Candidate Biomarkers	Ref.
*n* = 32	*n* = 32	LC–MS GC–MS	PCA PLS-DA	1. Glycine (−); Serine (−); Threonine (−); Alanine (−) 2. Glutamine (−); Isocitrate/Citrate (−); Aconitate (−); Succinate (−) 3. Sucrose (−); Sorbose (−); Arabinose (−); Arabitol (−); Inositol (−); Galactarate (−); Acetate (−); Propanoic acid (−); Propenoic acid (−); Butanoic acid (−) 4. Carnitines (−) 5. Sphingolipids (+)	1. Amino acid metabolism 2. Energetic metabolism 3. Carbohydrates metabolism 4. Long-chain FAs metabolism 5. Sphingolipid metabolism	-	[92]
*n* = 59	*n* = 43	GC–MS	RF LDA	1. 2,6-Dimethyl-7-octen-2-ol (−); 3-Octanone (−); 2-Octanone (−) 2. Pentanal (+)	1. Increased energy consumption 2. Inflammatory conditions via the excessive production of reactive oxygen species, known to induce lipid peroxidation	4-Biomarker panel: 2,6-Dimethyl-7-octen-2-ol 3-Octanone 2-Octanone Pentanal (accu: 63–65%)	[93]
*n* = 66	*n* = 88 (BPH) + *n* = 11 (cancer-free)	UPLC-MS/MS	ROC Student′s t-test	Spermine (−)	Polyamines synthesis	Spermine (AUC: 0.83)	[94]
*n* = 62	*n* = 42	LC-QTOF	PLS-DA Model Performance: Sens: 88%; Spec: 93%	1. Dimethyllysine (−); 5-Acetamidovalerate (−); Acetyllysine (−); Trimethyllysine (−) 2. Imidazole lactate (−); Histidine (−); Methylhistidine (−); Acetylhistidine (−) 3. Urea (−); Acetylarginine (−); Acetylcitrulline (−); Acetylputrescine (−); Dimethylarginine (−); Citrulline (−) 4. Tyrosine (−) 5. 8-Methoxykynurenate (−); Kynurenic acid (−); Xanthurenic acid (−) 6. Sulfoacetate (−); Isethionate (−); Acetyltaurine (−) 7. Acetylaspartylglutamic acid (−); Acetylaspartate (−); 2-Oxoglutaramate (−) 8. 2-Pyrrolidone-5-carboxylate (−) 9. 5-Methyldeoxycytidine-5′-phosphate (−); 7-Methylguanosine (−); 7-Methylguanine (+)	1. Lysine degradation 2. Histidine degradation 3. Arginine metabolism 4. Tyrosine metabolism 5. Tryptophan metabolism 6. Taurine metabolism 7. Alanine, aspartate and glutamate metabolism 8. Glutamine and glutamate metabolism 9. Purine and pyrimidine metabolism	-	[95]
*n* = 30 Validation set *n* = 19	*n* = 25 Validation set *n* = 15	LC-ESI-MS/MS	PLS-DA Model performance: Sens: 90% Spec: 73%	1. Taurine (+) 2. Ethanolamine (−); Phosphoethanolamine (−) 3. Arginine (−); Homocitrulline (−); Citrulline (−) 4. Isoleucine (−); Leucine (−); Phenylalanine (−); Serine (−); Tyrosine (−); Tryptophan (−); Asparagine (−); Glutamate (−); Ornithine (−); Glutamine (−) 5. Lysine (−); δ-Hydroxylysine (−) 6. 1-Methylhistidine (−); 3-Methylhistidine (−); Histidine (−) 7. α-Aminoadipic acid (−); γ-Amino-*n*-butyric acid (−) 8. Cystathionine (−); Cystine (−); Methionine (−)	1. Energetic metabolism 2. Phospholipid metabolism 3. Arginine metabolism 4. Amino acid metabolism 5. Lysine degradation 6. Histidine degradation 7. FAs metabolism 8. Methionine metabolism	γ-Amino-n-butyric acid (AUC: 0.93) Phosphoethanolamine (AUC: 0.88) Ethanolamine (AUC: 0.86) Homocitrulline (AUC: 0.84) Arginine (AUC: 0.83) δ-Hydroxylysine (AUC: 0.80) Asparagine (AUC: 0.77)	[96]
*n* = 64	*n* = 51 (BPH)	^1^H-NMR	OPLS-DA	1. Branched-chain amino acids (+); Glutamate (+); Glycine (−); Dimethylglycine (−) 2. 4-Imidazole-acetate (−) 3. Fumarate (−) 4. Pseudouridine (+)	1. Amino acid metabolism 2. Histidine metabolism 3. TCA cycle 4. RNA synthesis	-	[97]
*n* = 29	*n* = 21 (BPH)	HS-SPME-GC-MS	Shapiro–Wilks test, Levene′s test, ANOVA, Kruskal–Wallis test, Pearson test	Before prostate massage: 1. 3,5-Dimethylbenzaldehyde (−) 2. 2,6-Dimethyl-7-octen-2-ol (−); 2-Ethylhexanol (−) 3. Santolin triene (−) 4. Furan (+) After prostate massage: 2. 3-Methylphenol (+); Phenol (+) 4. Furan (+) 5. 2-Butanone (+) 6. *p*-Xylene (+)	1. Alcohols and FAs metabolism 2. Lipid metabolism 3. Energetic metabolism 4. FAs oxidation 5. FAs and carbohydrate metabolism 6. Unavailable	Furan *p*-Xylene (correlation with GS)	[98]
*n* = 40 Validation set *n* = 18	*n* = 42 Validation set *n* = 18	GC–MS	PCA PLS-DA Model performance: Sens: 78% Spec: 94–100% Accu: 86–89% AUC: 0.90–094	1. Methylglyoxal (−) 2. Hexanal (−) 3. 3-Phenylpropionaldehyde (+); Decanal (−) 4. 4-Methylhexan-3-one (−); Hexan-2-one (−); 2-Methylcyclopentan-1-one (−); 5-Methylheptan-2-one (−); 4,6-Dimethylheptan-2-one (−);2-Hydroxy-2-methyl-1-phenylpropan-1-one (−); Pentan-2-one (+); Cyclohexanone (+) 5. 2.5-Dimethylbenzaldehyde (+) 6. 2,6-Dimethyl-6-hepten-2-ol (−);1-Methyl-4-propan-2-ylcyclohex-2-en-1-ol (−); Linalool (−); Terpinen-4-ol (−); 3- Carene (−); Isoterpinolene (−); Menthyl acetate (−); 7. Theaspirane (−) 8. Glyoxal (−) 9. 2-Butenal (−) 10. Phenylacetaldehyde (+) 11. Butan-2-one (+) 12. Dihydroedulan IA (−); 3,4-Dimethylcyclohex-3-ene-1-carbaldehyde (−); 4-Methyldec-1-ene (−); Hexadecane (+)	1. Pyruvate metabolism; Glycine, serine and threonine metabolism 2. Steroid hormone biosynthesis 3. Alcohols and FAs metabolism; Amino acids and carbohydrate catabolism 4. FAs metabolism 5. Alcohols and FAs metabolism 6. Lipid metabolism 7. Steroid metabolism 8. Energetic metabolism; metabolites related to cell signaling and membrane stabilization 9. Metabolites linked to lipid peroxidation 10. Phenylalanine metabolism 11. FAs and carbohydrate metabolism 12. Unavailable	6-Biomarker-panel: Hexanal 2,5-DiMethylbenzaldehyde 4-Methylhexan-3-one Dihydroedulan IA Methylglyoxal 3- Phenylpropional-dehyde (AUC: 0.90; sens: 89%; spec: 83%; accu: 86%)	[99]
*n* = 10	*n* = 30	GC–MS LC–MS	PCA OPLS-DA	1. Pseudouridine/Uridine (+); Dihydro-uridine (+) 2. Citrate (−) Pyruvate (+); Lactate (+); Hexose (−); Pentose (+) 3. Hippuric acid (−); Aminohippuric acid (+); Phenylpyruvic acid (−); Tyrosine (−) 4. Sphinganine (−); Sphingosine (−); Serine (+) 5. Succinate (−); Glucosamine phosphate (+) 6. Xanthosine (+); Hypoxanthine (+); Xanthine (+) 7. Hydroxytryptophan (+) 8. N-linoleoyl taurine (−); Taurine (+) 9. Creatinine (+) 10. Sialyl-N-acetyllactosamine (+); Suberic acid (+); Dihydrocaffeic acid sulfate (+); Hydroxyethanesulfonate (+); Hydroxyglutaric acid (+); Acetylaminoadipic acid (+);Adipic acid (+); Hydantoinpropionate (+); Nicotine glucuronide (−); Benzoic acid (−); Oxo-heptanoic acid (+); Glucoheptanic acid (−); Aminohexadecanoic acid (−); Glucocaffeic acid (−); Trimethyluric acid (+); 3,7-Dimethyluric acid (−); 3′ Sialyllactose (+)	1. Pyrimidine metabolism 2. Energetic metabolism (gluconeogenesis; pyruvate metabolism pathways; glycolysis; pentose phosphate pathway) 3. Phenylalanine metabolism 4. Sphingolipid metabolism 5. Alanine, aspartate and glutamate metabolism 6. Purine metabolism 7. Tryptophan metabolism 8. Taurine metabolism 9. Amino acid metabolism 10. Unavailable	-	[100]
*n* = 43	*n* = 48 (BPH)	GC–MS	PLS-DA PARAFAC2 Model performance: Sens: 93% Spec: 89%	1. Androsterone (+); 16-Hydroxydehydroisoandrosterone (+); 5β-Pregnanediol (−); Enterodiol (−); Pregnanetriol (−) 2. 5-Hydroxyindoleacetic acid (+) 3. Vanillyl alcohol (+)	1. Steroidal biosynthesis 2. Tryptophan metabolism 3. Unavailable	-	[101]
*n* = 41 Validation set *n* = 18	*n* = 42 Validation set *n* = 18	GC–MS ^1^H-NMR	PCA PLS-DA Model performance: GC–MS Sens: 89% Spec: 83%, Accu: 86% AUC: 0.96 ^1^H NMR Sens:67% Spec: 89% Accu:78% AUC: 0.82	1. Pyruvate (+); Leucine (+); Valine (+) 2. Gluconic acid (−); D-Glucose (−); D-Mannitol (−); D-Threitol (+); L-Fucitol (−); L-Threose (+) 3. Sarcosine (+); Hydroxyacetone (+); 2-Furoylglycine (−) 4. L-Arabitol (−); Ribitol (−) 5. Propylene glycol (+) 6. Acetone (+) 7. Trigonelline (−) 8. Oxalate (+) 9. *Myo*-inositol (−) 10. 2-Hydroxyisobutyrate (+); 2-Hydroxyvalerate (+)	1. Valine, leucine and isoleucine biosynthesis and degradation 2. Energetic metabolism (Pentose phosphate pathway; Glycolysis or gluconeogenesis) 3. Glycine, serine and threonine metabolism 4. Pentose and glucuronate interconversions 5. Pyruvate metabolism 6. Propanoate metabolism; Synthesis and degradation of ketone bodies 7. Nicotinate and nicotinamide metabolism 8. Glyoxylate and dicarboxylate metabolism 9. Galactose metabolism; Ascorbate and aldarate metabolism; Membrane metabolism 10. Unavailable	2-Hydroxyvalerate (sens: 86%; spec: 61%; AUC 0.76) 2-Furoylglycine (sens: 85%; spec: 62%; AUC 0.74) D-Glucose (sens: 70%; spec: 69%; AUC 0.69) D-Mannitol (sens: 78%; spec: 60%; AUC 0.69)	[102]
*n* = 20	*n* = 20 (cancer-free) *n* = 20 (bladder cancer) *n* = 20 (renal cancer)	GC–MS	PCA PLS-DA	1. Methylglyoxal (−) 2. Hexanal (−) 3. 3-Phenylpropionaldehyde (+) 4. 4-Methylhexan-3-one (−) 5. 2.5-Dimethylbenzaldehyde (+) 6. Dihydroedulan IA (−) 7. Ethylbenzene (+) 8. Heptan-2-one (+); Heptan-3-one (+); 4-(2-Methylpropoxy) butan-2-one (+) 9. Methyl benzoate (+) 10. 3-Methylbenzaldehyde (+)	1. Pyruvate metabolism; Glycine, serine and threonine metabolism 2. Steroid hormone biosynthesis 3. Alcohols and FAs metabolism; amino acid and carbohydrate catabolism 4. FAs metabolism 5. Alcohols and FAs metabolism 7. Metabolites linked to oxidative stress 8. Protein metabolism; Ketogenic pathway 9. Lipid hydrolysis 10. Metabolites linked to lipid peroxidation	10-biomarker panel Methylglyoxal Hexanal 3-Phenylpropionaldehyde 4-Methylhexan-3-one 2.5-Dimethylbenzaldehyde Dihydroedulan IA Ethylbenzene Heptan-2-one Heptan-3-one 4-(2-Methyl-propoxy)butan-2-one Methyl benzoate 3-Methylbenzaldehyde Discrimination of PCa from control, bladder cancer and renal cancer (AUC. 0.90; sens: 76%, spec: 90%, accu: 92%)	[103]
*n* = 58	*n* = 18 (BPH)	^1^H-NMR	PCA PLS-DA	1. Glutamate (−); Glutamine (−); Glycine (−) 2. Citrate (−); Taurine (−) 3. Trimethylamine (+) 4. Choline (−)	1. Amino acid metabolism 2. Energetic metabolism 3. Membrane metabolism 4. Choline metabolism; phospholipid membrane metabolism	Citrate Glutamine	[75]

Notes: (+) indicates increased levels in PCa, (-) indicates decreased levels in PCa; the numbering of the column *Altered Metabolites* is related with the numbering of the column *Dysregulated metabolic pathways*. Abbreviations: ^1^H-NMR, proton nuclear magnetic resonance spectroscopy; accu, accuracy; AUC, area under the curve; BPH, benign prostatic hyperplasia; FAs, fatty acids; GC–MS, gas chromatography–mass spectrometry; GS, Gleason score; HS-SPME, headspace solid-phase microextraction; LC-ESI-MS/MS, liquid chromatography electrospray ionization tandem mass spectrometry; LC–MS, liquid chromatography–mass spectrometry; LC-QTOF, liquid chromatography quadrupole time of flight; LDA, linear discriminant analysis; OPLS-DA, orthogonal projections to latent structures discriminant analysis; PCA, principal component analysis; PLS-DA, partial least squares-discriminant analysis; RF, random forest; ROC, receiver operating characteristics curve; sens, sensitivity; spec, specificity; TCA, tricarboxylic acid cycle; UPLC-MS/MS, ultra-performance liquid chromatography-tandem mass spectrometry.
